# DC/L‐SIGNs of hope in the COVID‐19 pandemic

**DOI:** 10.1002/jmv.25980

**Published:** 2020-06-19

**Authors:** Adam Brufsky, Michael T. Lotze

**Affiliations:** ^1^ Department of Medicine, Division of Hematology‐Oncology, School of Medicine, UPMC Hillman Cancer Center, Magee Women's Hospital University of Pittsburgh Medical Center Pittsburgh Pennsylvania; ^2^ Department of Surgery, UPMC Hillman Cancer Center University of Pittsburgh Medical Center Pittsburgh Pennsylvania

In a rapidly evolving pandemic such as COVID‐19, theories which help unify disparate pre‐clinical and clinical observations would be useful. The current pandemic and its pleotropic effects can be explained in part by interaction between SARS‐CoV‐2 spike protein S, the ACE2/L‐SIGN/CD209 receptor on the type II alveolar cell of the lung, and the DC‐SIGN receptor on the respiratory dendritic cell (DC) and associated endothelial cells. Infection of the DC by SARS‐CoV‐2 can potentially explain the exuberant distal immunopathology seen in COVID‐19.

In a mouse model of severe acute respiratory distress syndrome (SARS), the human disease most related to coronavirus disease 2019 (COVID‐19) in clinical course, severe disease is correlated with slow kinetics of SARS coronavirus (CoV) viral clearance.^1^ This is accompanied by delayed activation and transit of respiratory dendritic cells (DCs) to the draining lymph nodes with a deficient virus‐specific T‐cell response.^1^ In the SARS‐CoV‐2 infection, there is initial lymphopenia, and the lymphocyte count is predictive of disease severity and mortality.^2^ Lymphocyte counts recover with viral clearance and disease resolution, with adaptive immune cells (CD3+ T‐cells) being especially important.^2^ Such immune deficiency can in part be explained by viral infection and T‐cell interaction with the respiratory DC.

Early and central infection of tissue‐resident DC by the SARS‐CoV‐2 coronavirus explain some of the immunopathology of the COVID‐19 pandemic. DC are richly abundant in the lung and responsive to viral infection.^3^ RNA expression profiling studies demonstrate that human DC express the angiotensin‐converting enzyme 2 (ACE2) receptor for SARS‐CoV‐2.^4^ In COVID‐19, T‐cell receptor (TCR) repertoires are dramatically reduced during the early onset of severe SARS‐CoV‐2 infection but recover during the convalescent stage.^5^ Such reduction of T‐cells suggest acute wholesale apoptotic death with engagement of the TCR in the absence of costimulatory molecules, normally provided by DC.^6^

DC also express DC‐SIGN. Dendritic cell‐specific ICAM‐3‐grabbing non‐integrin (DC‐SIGN) is a membrane receptor of a C‐type lectin family expressed on DCs with a primary function of recognizing high mannose glycans present on other cellular receptors or pathogens.^7^ With L‐SIGN (CD209L, DC‐SIGNR, or liver/lymph specific SIGN) expression, the other SIGN found in humans,^8^ these mannose receptors are involved in virus capture and entry into cells.^9^ L‐SIGN is expressed on human type II alveolar cells, is associated with ACE2,^10^ and can enhance ACE2 mediated binding and cellular entry of viral pseudotypes expressing the spike protein S of SARS‐CoV.^9^

Lentiviral pseudotyped viruses expressing SARS‐CoV S protein require acidification of the endosome for viral entry.^11^ DC‐SIGN mediates binding of these pseudotyped vectors to human DC with uptake into the endosome, followed by polarization of the endosome and delivery of the virus in an “infectious synapse.”^11^ This appears similar to an infectious synapse between infected DC and T cells that facilitates HIV infection mediated by DC‐SIGN.^12^

Deglycosylation reduces infectivity of viral pseudotypes expressing SARS‐CoV spike protein^13^ and specific asparagine glycosylation sites in three clusters within the SARS‐CoV S protein appear critical for DC/L‐SIGN mediated, but not ACE2 mediated, SARS Co‐V pseudotype entry into cells.^13^ Infectivity mediated through DC/L‐SIGN is reduced in proportion to the number of mutated glycosylated sites, suggesting that the number of glycosylated sites, and not just specific mutation, is important.^13^ Comparison of amino acid sequences of the S protein in SARS‐like coronavirus in civets as well as in several human SARS‐CoV isolates demonstrates progressive mutation of additional NXS/T amino acid canonical glycosylated sites in the human strains.^13^ This suggests a mechanism for increase in viral virulence through progressive glycosylation of the SARS‐CoV‐2 spike.

This may explain a recently reported observation where a stable nonsynonymous D614G mutation in the spike glycoprotein of SARS‐CoV‐2 appears to arise from an ancestral aspartic acid (D) residue early in the course of the pandemic.^14^ This ancestral D appears to be more common in the West Coast of the United States, and the glycine (G) residue is more common in the East Coast, where substantial differences in mortality and transmission are observed. The D614G mutation is predicted to be present in a highly glycosylated portion of the viral spike two amino acids N‐terminal to a predicted NXS/T glycosylated asparagine at residue 616.^15^ Statistical analysis of the protein environment of N‐glycosylation sites^16^ suggests that replacement of a polar D by an aliphatic G at residue 614 should increase the probability of glycosylation of the asparagine NXS/T site at residue 616. This is predicted to increase virulence of SARS‐CoV‐2 through increased glycosylation at that site. Increased binding of this G variant to either DC‐ or L‐SIGN in both type II alveolar cells, as well as DCs, could explain in part the observed mortality and transmissibility differences.

Several reports^17^, ^18^ now suggest that there are multiple SARS‐CoV‐2 isolates with stable mutations in the spike S protein. SARS‐CoV‐2 mutations described one of these recent reports^17^ (Table 1) demonstrates that these are either predicted to increase or decrease glycosylation at various sites in the in the spike S protein, and these may possibly account for differences in virulence. A stable mutation in the SARS‐CoV‐2 spike protein found in one report is predicted to bind ACE2 less tightly, predicting a decrease in virulence. SARS‐CoV‐2 strains with various mutations in the viral spike can vary as much as 270‐fold in virulence in Vero E6 culture.^18^ Viral evolutionary theory suggests that one option for a viral strain introduced to a novel host is to maintain fitness through reduction in virulence,^19^ and these various glycosylation mutations in the spike protein can plausibly provide one such mechanism for attenuation.

**Table 1:** Analysis of spike protein S mutations in six viral strains from Jia et al^17^

Mutation	Place and date	Predicted spike protein function	Predicted activity
R408I	India 27‐01‐20	Decrease ACE2 binding	Attenuation
D614G	CA, 11‐03‐20	Increase glycosylation at aa 616	Virulence
A28N	China 17‐01‐20	New glycosylation site at aa 28	Virulence
W655Y	WA, 01‐03‐20	Decrease glycosylation at aa 657	Attenuation
F797C	Sweden 07‐02‐20	Decrease glycosylation at aa 801	Attenuation
N74K	China 20‐01‐20	Eliminate glycosylation site at aa 74	Attenuation

A single‐nucleotide polymorphism in the promoter region of the DC‐SIGN gene is associated with disease severity in SARS.^20^ This underscores the likely involvement of DC/L‐SIGN family members in the pathogenesis, virulence, and attenuation of the pathogen responsible for SARS and most likely COVID‐19. Strategies designed to limit T‐cell apoptosis through DC modulation, limiting expression or glycosylation of SARS‐CoV‐2 spike protein, or possibly driving intracellular degradation of viral proteins, should be considered.

## CONFLICT OF INTERESTS

The authors declare that there are no conflict of interests.
